# MmpL12 transports lipooligosaccharides and impacts virulence in Mycobacterium marinum

**DOI:** 10.1099/mic.0.001618

**Published:** 2025-10-08

**Authors:** Rebeca Bailo, C. M. Santosh Kumar, Albel Singh, Peter A. Lund, Vassiliy N. Bavro, Apoorva Bhatt

**Affiliations:** 1 School of Biosciences and Institute of Microbiology and Infection, University of Birmingham, Edgbaston, Birmingham B15 2TT, UK; 2 School of Life Sciences, University of Essex, Colchester, CO4 3SQ, UK

**Keywords:** lipooligosaccharide, lipid transport, *Mycobacterium*, resistance-nodulation-division (RND) family

## Abstract

Lipooligosaccharides (LOSs) are polar glycolipids found in the cell envelope of many pathogenic mycobacteria. Here, we show that LOS transport in *Mycobacterium marinum* requires *mmpL12*, a member of the resistance-nodulation-division family of membrane proteins. Deletion of *mmpL12* resulted in a rough colony morphology and increased hydrophobicity. The △*mmpL12* mutant accumulated three of the biosynthesis intermediates of LOSs (LOS-I, LOS-II and LOS-III) intracellularly and failed to produce the final product, LOS-IV, suggesting that final glycosylation of LOS-III to yield LOS-IV occurs extracellularly after LOS-III export. *In silico* structural analysis of the MmpL12 suggests that it is a proton-driven transporter that shares very similar organization with other subclass 1 MmpLs (MmpL1, 2, 4–8 and 9–10), featuring a large periplasmic loop (PD3 domain) which is predicted to form a large coiled coil that may be involved in the trimerization of this subset of MmpL transporters. Furthermore, the long C-terminal extension domain, which is unique to MmpL12, may provide additional trimerization support and scaffold for assembly of additional LOS biosynthetic enzymes. The absence of any extracellular LOS intermediates and of LOS-IV had an impact on virulence, with the mutant strain exhibiting a larger bacterial burden in infected zebrafish embryos.

## Introduction

**Fig. 1.**
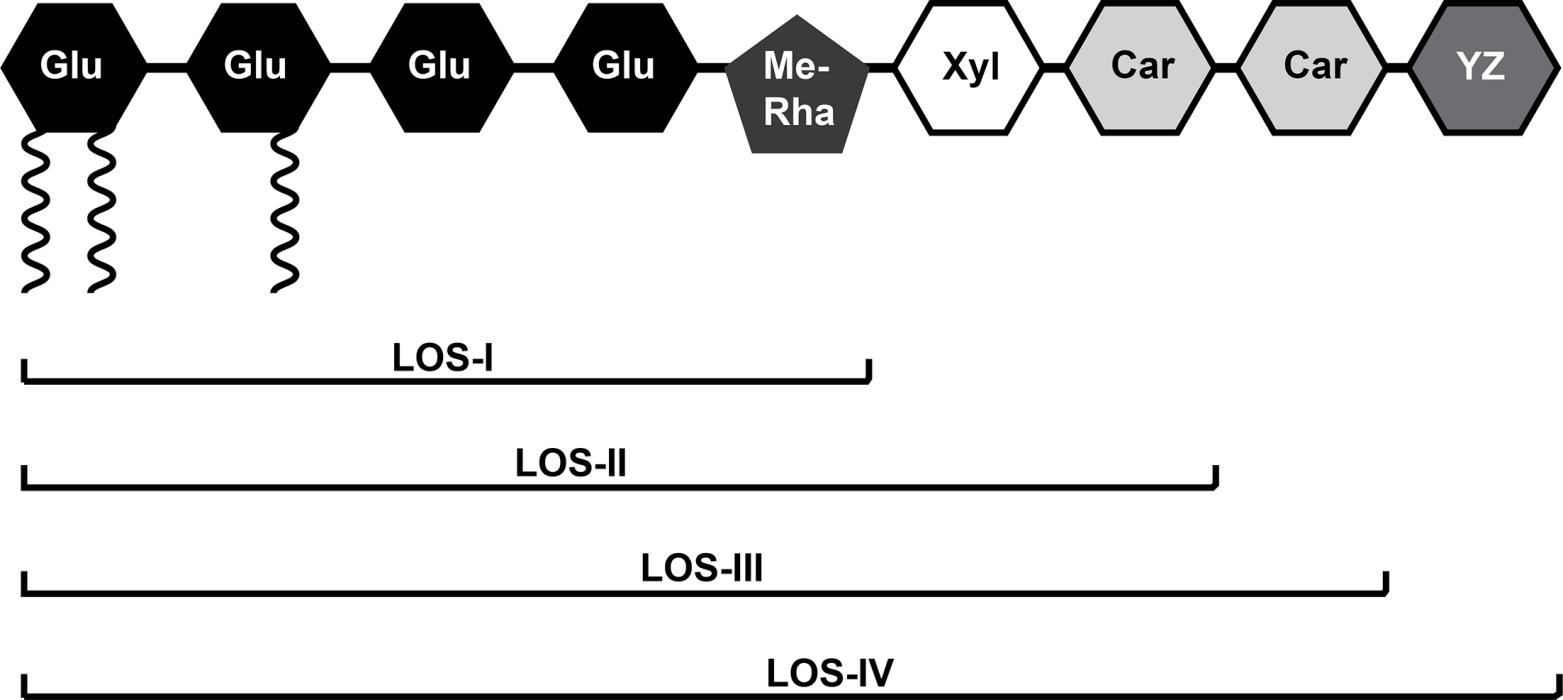
Schematic representation of LOSs produced by *M. marinum*. Glu, glucose; Me-Rha, methyl rhamnose; Xyl, xylose; Car, caryophyllose; YZ, pyrrolidone-substituted dideoxy galactose. Acyl chains are shown as black coils.

Bacterial species of the genus *Mycobacterium* have a unique, lipid-rich cell envelope that is responsible for resistance to antimicrobials, and in the case of pathogens, such as the tuberculosis bacillus (*Mycobacterium tuberculosis*), plays a role in immunomodulation and virulence [1,3]. Included in this list of complex and distinct lipids are a class of glycolipids termed lipooligosaccharides (LOSs). LOSs are found in many pathogenic mycobacterial species, including *Mycobacterium kansasii*, *Mycobacterium gastri*, *Mycobacterium szulgai*, *Mycobacterium marinum* and *Mycobacterium canetti* (the smooth-surface variant of *M. tuberculosis*). Interestingly, *M. tuberculosis* has undergone reductive evolution and contains approximately a third of genes found in *M. canetti* and consequently does not produce LOSs. The polar nature of LOSs has led to the hypothesis that these glycolipids likely played a role in the transmission and evolution of the tubercle bacillus as a human pathogen [4,5]. The role of LOSs in mycobacterial biology has been predominantly studied in *M. marinum*, which produces four subclasses of LOSs [6,7], LOS-I, LOS-II, LOS-III and LOS-IV, each containing a common acylated glycan core consisting of four glucose residues and one methylated rhamnose (Fig. 1). LOS-II, LOS-III and LOS-IV all contain a d-Xyl*p* (xylopyranose) residue in addition to the glycan core, with each subclass further elaborated by novel sugar residues [8,9]. In other words, LOS-I, LOS-II and LOS-III are intermediates in the biosynthesis of LOS-IV. The gene cluster responsible for LOS biosynthesis in *M. marinum* was first identified by transposon mutagenesis and contains genes encoding glycosyl transferases and those encoding enzymes required for the biosynthesis and transfer of the fatty acyl components of LOS [7]. Complex cell wall glycolipids in mycobacteria are primarily exported by transmembrane (TM) proteins that belong to the resistance-nodulation-division (RND) of transporters and are termed MmpLs (mycobacterial membrane protein large) [10,11]. The gene cluster responsible for the biosynthesis of LOSs in *M. marinum* also has an *mmpL* gene, *MMAR_2342* (UniProt ID B2HQ48), orthologous to *M. tuberculosis mmpL12*. Due to its association with the LOS biosynthesis cluster, it was likely that *MMAR_2342* (hereby referred to as *M. marinum mmpL12*) was responsible for the export of LOSs in *M. marinum*. To probe this potential role of *mmpL12*, we generated an *M. marinum mmpL12* null mutant to assess the effects on LOS transport in the mutant. The mutant afforded us an opportunity to query whether *mmpL12* was responsible for the export of fully synthesized LOS (LOS-IV), or the transport of a biosynthesis intermediate that was further glycosylated outside the cell envelope. Additionally, we also used the mutant to assess the impact of loss of *mmpL12* on virulence in a zebrafish embryo model of infection. Finally, to gain some functional insights into the possible organization of the MmpL12, we also did some *in silico* structural analysis, which revealed some unexpected similarities with other MmpLs and suggests a novel mode of MmpL subclass 1 oligomerization.

## Methods

### Bacterial strains and growth conditions

WT (*M. marinum* 1218R; ATCC 927), △*mmpL12* mutant and complemented strains were routinely grown at 30 °C in Middlebrook 7H9 broth (BD) supplemented with 0.05% Tween 80 and 0.2% glycerol and on Middlebrook 7H10 plates (BD) supplemented with 10% Middlebrook OADC (oleic acid/albumin/dextrose/catalase) (BD) and 0.5% glycerol. *Escherichia coli* TOP10 (Invitrogen) were cultured in Luira-Bertani (LB) broth (BD) and LB agar prepared by adding 1.5% agar to LB broth prior to autoclaving. The antibiotic concentrations used for *M. marinum* were 25 µg ml^−1^ for hygromycin (Invitrogen), 25 µg ml^−1^ kanamycin (Sigma-Aldrich) and 50 µg ml^−1^ apramycin (Sigma-Aldrich), and for *E. coli* were 100 µg ml^−1^ for hygromycin and 50 µg ml^−1^ kanamycin.

### Construction of the *mmpL12* deletion mutant and complemented strain

A null mutant of *M. marinum* 1218R was constructed using specialized transduction [12,13]. Briefly, a derivative of temperature-sensitive phage phAE159 containing the *mmpL12* (*MMAR_2342*) upstream and downstream regions flanking a hygromycin cassette (phΔ*mmpL12*) was generated. Primers used for flanking sequence amplifications were MM2342LL (5′ TTTTTTTTCCATAAATTG GTGGCAAACGTGCCGTTTGAG 3′), MM2342LR (5′ TTTTTTTTCCATTTCTTGG TTTGTGTACGCGGCCAAGTC 3′), MM2342RL (5′ TTTTTTTTCCATAGATTGG ACACCAACGGCAACGGCAAC 3′) and MM2342RR (5′ TTTTTTTTCCATCTTTT GGTCTTCCGTGCGCGGATAGTC 3′). After selection of phΔmmpL12 transductants in *M. marinum* 1218R (WT) at 37 °C, one hygromycin-resistant colony was verified by whole-genome sequencing to have the *mmpL12* gene replaced by the hygromycin resistance cassette. This transductant was named Δ*mmpL12* and used in all subsequent experiments. The complemented strain, Δ*mmpL12*-C, was obtained by transformation of pMV2342 in Δ*mmpL12* mutant. pMV2342 was created by cloning *M. marinum mmpL12* with its native promoter in a single-copy, integrating vector, pMV306 [14] using the primers Mmar2342for 5′ GCGTCTAGACTAGTAGGGGGCCGCAAC 3′ and Mmar2342rev 5′ GCGAAGCTTCAATTGAGGTGTGGCTGC 3′.

### Construction of *M. marinum* strains for zebrafish infections

To visualize *M. marinum* strains during the zebrafish infections, mWasabi from pTEC15 [15] was released using NdeI and NheI and cloned in pMSP12-dsRED2 [16]). Previously, the NheI restriction site was introduced by site-directed mutagenesis in pMSP12-dsRED2 using the primers dsRED_Nhe_F 5′ CCACCTGTTCCTGTAGGCTAGCGCGACTCTAGCCACC 3′ and dsRED_Nhe_R 5′ GGTGGCTAGAGTCGCGCTAGCCTACAGGAACAGGTGG 3′. The resulting vector, pSK100, was transformed in the Δ*mmpL12* mutant. A fluorescent Δ*mmpL12*-C strain was generated by transforming it with an apramycin-resistant version of pSK100, called pRB100. The modification consisted of replacing the kanamycin cassette in pSK100 by the apramycin cassette from pMV261apra [17] using the primers pSK100_Fw 5′ TCAGAATTGGTTAATTGGTTG 3′, pSK100-apra 5′ AACACCCCTTGTATTACTG 3′, pSK100_apra_Fw 5′-GCTTACATAAACAGTAATACAAGGGGTGTTATGTCGTG CAATACGAATGGCGAAAAG-3′ and pSK100_apra_Rv 5′ CCAGTGTTACAACCAA TTAACCAATTCTGATCAGCGCGACCTTGCCCC 3′ and the Gibson cloning method to create the pRB100 vector. pMSP12::dsRed2 (Addgene plasmid # 30171; http://n2t.net/addgene:30171; RRID:Addgene_30171) and pTEC15 (Addgene plasmid # 30174; http://n2t.net/addgene:30174; RRID:Addgene_30174).

### Determination of hydrophobicity

Hydrophobicity measurement of *M. marinum* strains using hexadecane partitioning was done as described before [5]. Briefly, liquid culture grown in supplemented 7H9 was resuspended and washed twice in Phosphate Urea Magnesium (PUM buffer) and finally resuspended in the buffer to an A_600_ of 0.7. Aliquots (3 ml) were transferred to glass tubes and mixed with 2.4 ml of hexadecane for 1 min. Samples were further incubated for 8 min at 37 °C and then at 22 °C for 15 min until the two phases had separated. A_600_ of the aqueous phase was measured. The hydrophobicity index was expressed as a percentage of that of the bacterial suspension in PUM buffer alone to the aqueous phase reading. Results were expressed as mean sd of three independent experiments. Plate cultures in the presence of Congo red were done using 7H10 agar plates containing 10% OADC, 0.5% glycerol and 100 µg ml^−1^ Congo red (from a 50 mg ml^−1^ filter sterilized aqueous stock solution). Ten microlitres of aliquots from mid-log phase cultures of *M. marinum* strains were spotted on the plates and at 30 °C for 1 week.

### Lipid analysis of *M. marinum* strains

*M. marinum* 1218 R WT, Δ*mmpL12* and Δ*mmpL12*-C strains were grown in 20 ml 7H9 broth, and lipids were labelled by adding 0.5 µCi ml^−1^ [^14^C] acetate (45–60 mCi/mmol) (PerkinElmer) at late log phase for a period of 48 h. Lipids from these cultures were obtained in three fractions as described by Dobson *et al*. [18], with a few modifications. Briefly, outside apolar and polar lipids from dried pellets were extracted with 2 ml of water-saturated *n*-butanol in two consecutive extractions and dried. Next, inner apolar and polar lipids were extracted following the protocol described by Dobson *et al*. [18]. The extracted lipid fractions were solubilized in chloroform:methanol (2 : 1 v/v) mixture. Aliquots (15,000 c.p.m.) from each outside and inside polar lipid extracts were analysed by 2D-TLC utilizing Silica Gel 60 F254 plates (Merck) developed once in the solvent system CHCl_3_/CH_3_OH/H_2_O (60 : 30 : 6, v/v/v) in the first direction and once in the solvent system CHCl_3_/CH₃COOH/CH_3_OH/H_2_O (40 : 25 : 3 : 6, v/v/v/v) in the second direction. Autoradiograms were produced after exposing Carestream Kodak BioMax MR film for 7 and 3 days for the outside and inside polar lipids, respectively.

### Zebrafish embryo infections

All the infection experiments were performed, in accordance with the respective UK Home Office guidelines and regulations, on zebrafish larvae up to 5 days post-fertilization. The transgenic zebrafish line, Tg (mpeg1:G/U:NfsB-mCherry) with macrophage-specific expression of mCherry, was used for the infections. After the collection of eggs, larvae were incubated at 28 °C in 1× E3 media (5 mM NaCl, 0.17 mM KCl, 0.33 mM CaCl_2_, 0.33 mM MgSO_4_, pH 7 and 0.00003% methylene blue). The medium was changed every 2 days. The fish were anaesthetized with 200 µg ml^−1^ buffered tricaine (3-aminobenzoic acid ethyl ester) (Sigma-Aldrich) during bacterial injections and imaging.

*M. marinum* strains were prepared for the infections following standard protocols [19,20]. Briefly, *M. marinum* WT, *ΔmmpL12* and *ΔmmpL12*-C strains, expressing mWasabi, were cultured statically at 30 °C for 48 h in Middlebrook 7H9 broth supplemented with 10% Middlebrook OADC and appropriate antibiotics. The cultures were harvested at mid-log growth (OD_600nm_ = 0.7–1). OD_600nm_ 1 represents 10^8^ c.f.u. ml^−1^. We attempted to break bacterial clumps in the Δ*mmpL12* culture by passing the culture through a 27-gauge needle multiple times. Harvested bacteria were washed three times in PBS and resuspended in 2% polyvinylpyrrolidone (PVP-40) (Sigma-Aldrich)/PBS containing 10% phenol red (Sigma-Aldrich) to aid visualization of injections.

Hindbrain infections were conducted as previously described [21]. Transgenic zebrafish were screened at prim-25 stage [22], and only larvae expressing the mCherry fluorescent protein were selected for injection. The embryos staged at 24 h post-fertilization (hpf) were manually dechorionated using jeweller’s forceps (Dumont #5, World Precision Instruments Inc., Sarasota, FA, USA), anaesthetized at 28–30 hpf with 160 µg ml^−1^ Tris-buffered tricaine methane sulfonate (Tricaine; Sigma-Aldrich) and infected by micro-injection of ~200 c.f.u. of *M. marinum* strains into the hindbrain ventricle. Fifty embryos were infected with each strain separately. Control embryos were injected with PVP. Survival was monitored every 24 h under the light microscope by observing the heartbeat of the larvae. The survival was compared to that of the uninfected control larvae.

Live, anaesthetized larvae were imaged 4 days post-infection (dpi) at 20× on a Nikon Eclipse Ti Live stereo fluorescence microscope equipped with a QICAM 607 Fast 1394 CCD camera (Teledyne QImaging, Surrey, BC, Canada). Twelve embryos per *M. marinum* strain were selected randomly for imaging. Brightfield and fluorescence images were captured as Z-series image stacks. Images were analysed using ImageJ [23] and QuantiFish [20] to quantify the bacterial burden and macrophage response as green and red fluorescent pixels, respectively.

### Molecular modelling

**Fig. 2.**
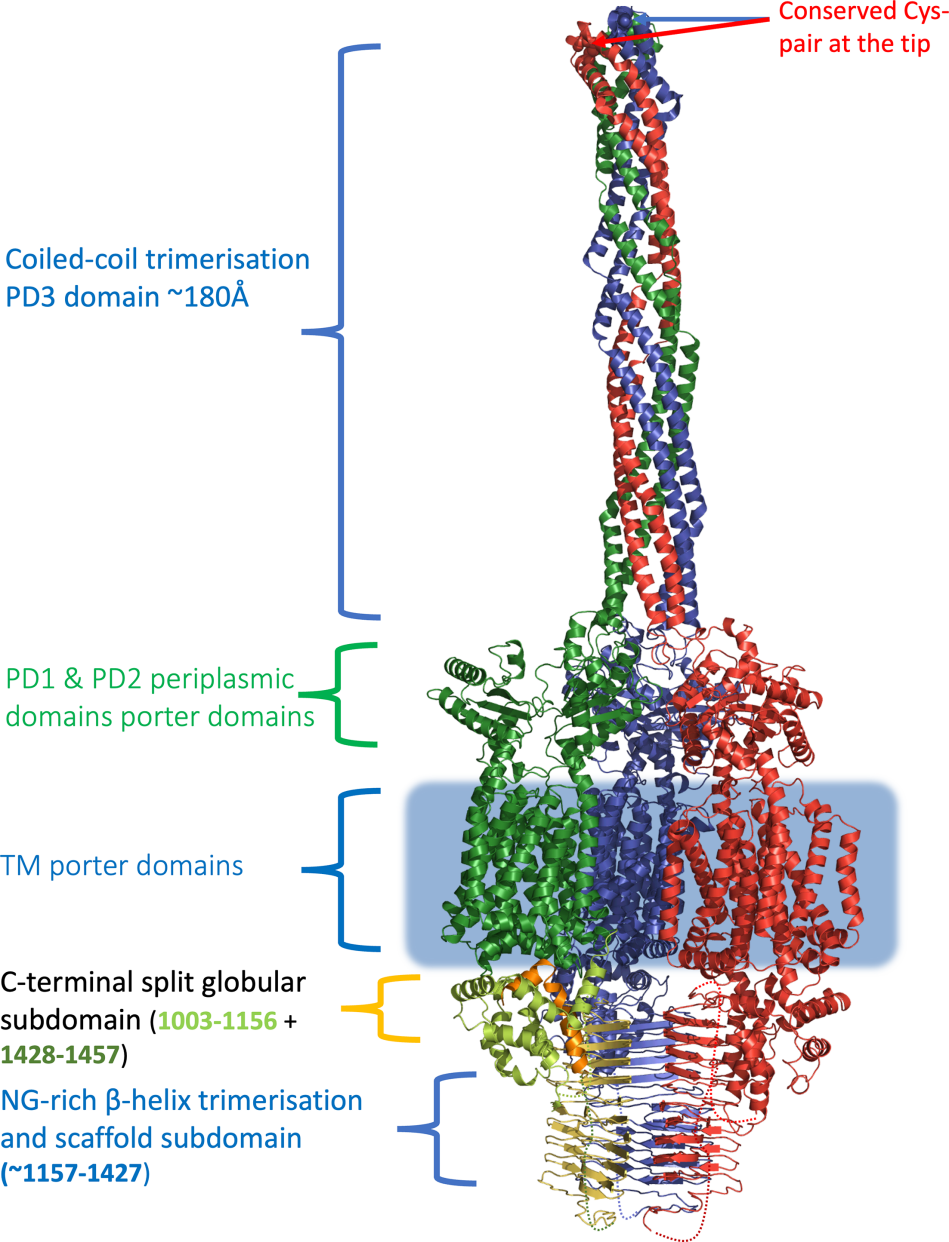
Qualitative composite model of the trimeric *M. marinum* MmpL12, based on AlphaFold 3, with individual chains coloured green, red and blue, respectively. Notably, the PD3 coiled-coil domains of all three subunits are suggested to form an extended periplasmic tube, providing a possible substrate conduit and playing a key role in trimerization of the MmpL12. While much of the C-terminal domain is predicted as a low complexity region in the monomeric model (see Figs S2 and S7), it appears to get more structured when MmpL12 was modelled as a trimer. We propose that part of the C-terminal domain may participate in the trimerization; however, it has to be noted that the model confidence in this region is low due to the lack of sequence coverage (see text and Fig. S7 for details).

AlphaFold 3 [24] (https://alphafoldserver.com/) was used for the majority of the modelling, including the oligomer mode. All models were generated with at least three technical repeats, with different seed per run and five predictions per seed. Predicted aligned error (PAE) matrix analysis and pLDDT visualization were performed using the AlphaFold3 server outputs and PAE webserver (https://pae-viewer.uni-goettingen.de/). Maximum multiple sequence alignment (MSA) depth analysis was provided via ColabFold v1.5.5: AlphaFold2 using MMseqs2 [25]. Average pLDDT scores were calculated using FirstGlance in Jmol (http://FirstGlance.Jmol.Org). Jmol is an open-source Java viewer for chemical structures in 3D (http://www.jmol.org). Structural analyses were performed using the DALI server [26]. Multiple sequence alignments were done with MAFFT [27], and structural alignments were visualized with Espript3 [28]. Molecular visualization performed using Pymol (the PyMOL Molecular Graphics System, v3.0 Schrödinger, LLC) and qualitative docking for Fig. 2 has been performed manually using Coot [29]. UniProt sequences used are indicated in the text.

## Results

### Deletion of *mmpL12* results in a strain with a rough colony morphology

**Fig. 3.**
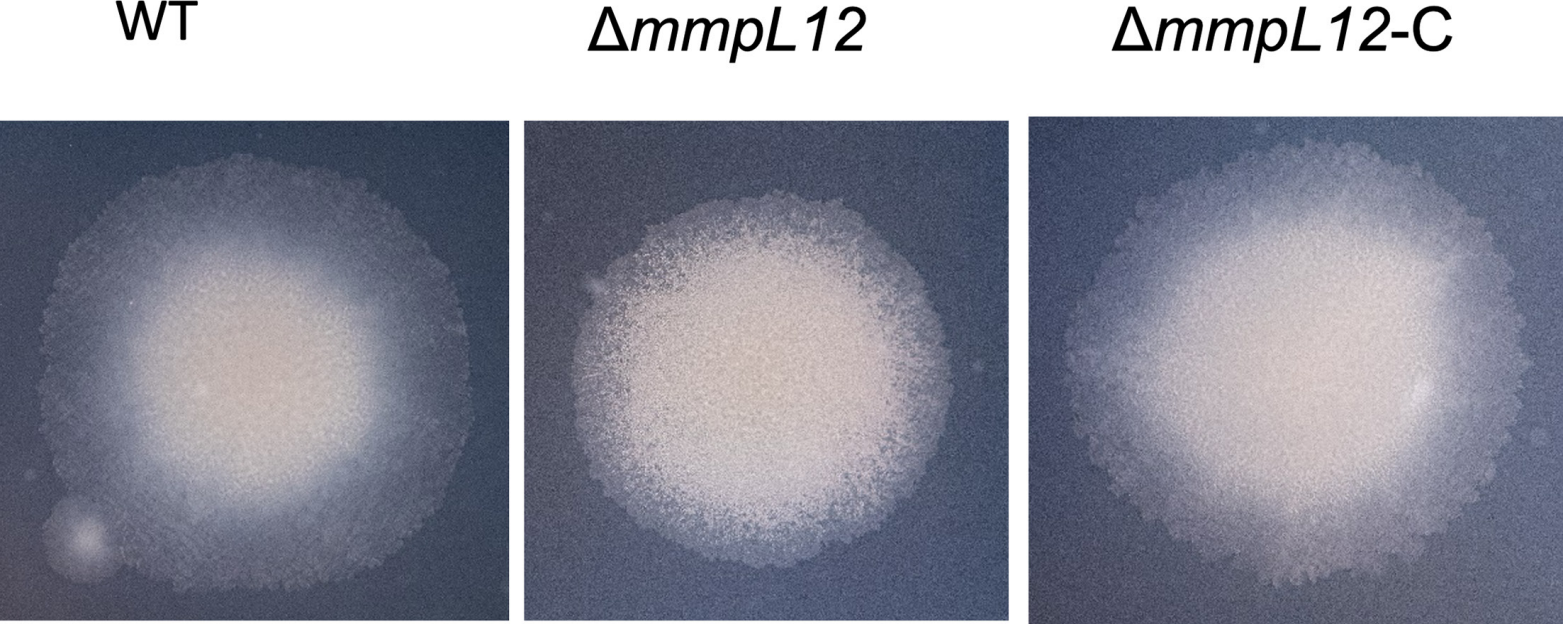
Colonies of *M. marinum* wild-type (WT), mutant (Δ*mmpL12*) and complemented (Δ*mmpL12*-C) strains on 7H9+ agar plates obtained after incubation at 30 °C for 1 week.

We used specialized transduction [12,13] to replace *mmpL12* in *M. marinum* 1218R (ATCC 927; referred to as WT) with a hygromycin resistance cassette (*hyg*); hygromycin-resistant, transduced strains were confirmed as bona fide *mmpL12* null mutants using whole-genome sequencing and referred to as △*mmpL12*. Colonies of the mutant strain showed a rough appearance when compared to the parental WT strain (Fig. 3). Colony morphology of the △*mmpL12* mutant was restored to that of the WT strain following the introduction of a single-copy integrative plasmid expressing *mmpL12* from its native promoter (△*mmpL12*-C), confirming that the observed change in colony morphology was solely due to the deletion of *mmpL12* (Fig. 3). A rough appearance in *M. marinum* colonies is usually associated with the absence of LOSs in the outer cell envelope [7], suggesting a defect in LOS transport in the △*mmpL12* strain.

### The △*mmpL12* mutant shows increased hydrophobicity

**Fig. 4.**
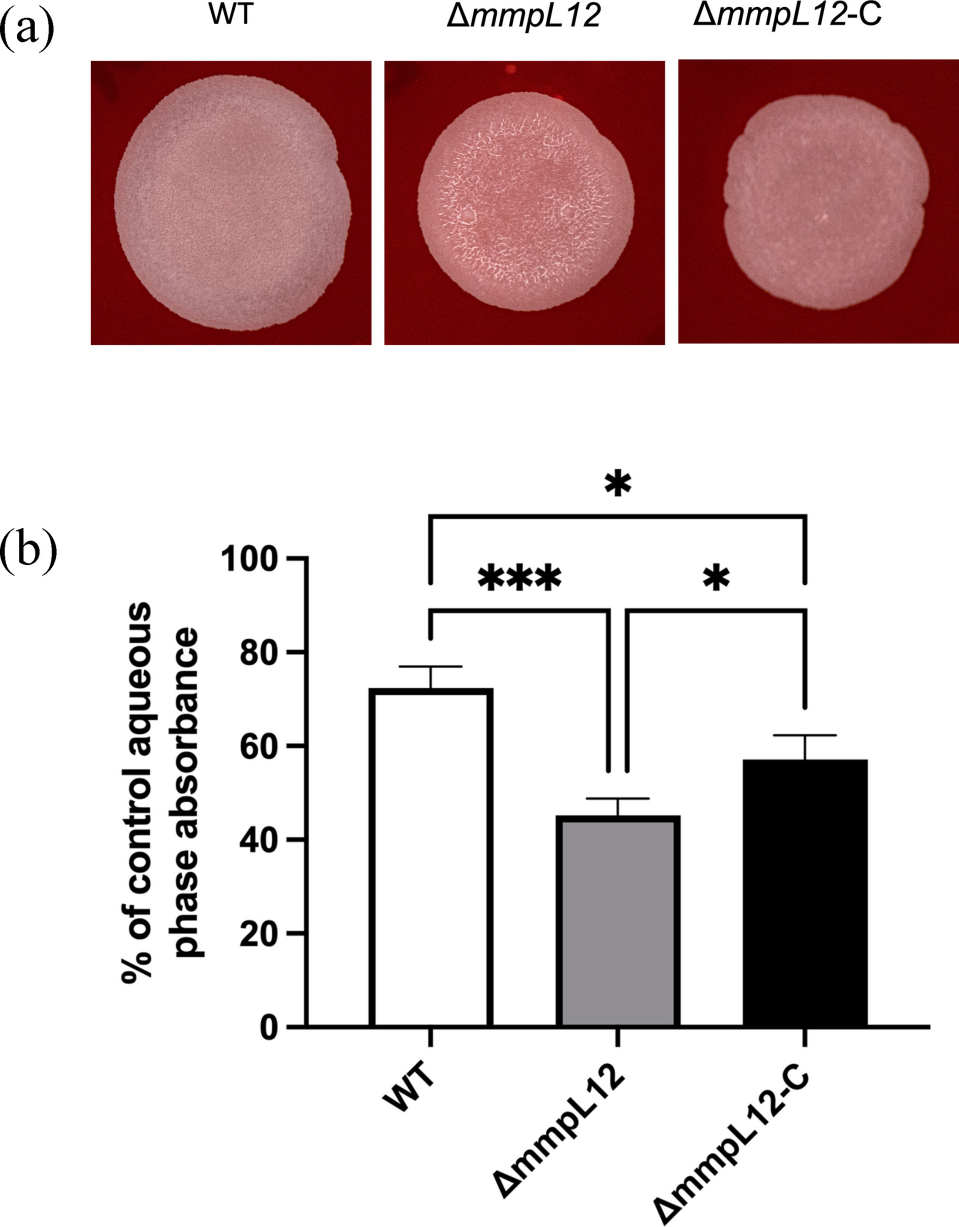
Effects of loss of *mmpL12* on *M. marinum* hydrophobicity as assessed by dye retention on Congo red-containing agar plates (**a**) and partitioning in hexadecane and water (**b**). *P<0.001, ***P<0.05.

The absence of the polar extracellular LOSs would be expected to increase the overall relative hydrophobicity of the mutant strain. We measured the hydrophobicity of the mutant strain, relative to the WT and △*mmpL12*-C, by two different methods. The first involved the use of Congo red, a dye that associates with lipophilic regions of the cell envelope and thus acts as an indirect measure of hydrophobicity. Cultures of the △*mmpL12* strain took up more of the dye, as shown by the darker red colonies formed by the mutant when grown on agar plates containing Congo red, compared to the WT and △*mmpL12*-C strains (Fig. 4a). We also measured the ability of all three strains to adhere to hexadecane in a two-phase aqueous hexadecane system. In this system, increased hydrophobicity correlates with increased retention in the hydrocarbon phase [5,30]. We observed increased retention of the △*mmpL12* strain in hexadecane (Fig. 4b), consistent with the dye retention outcome. These data showed that the loss of *mmpL12* resulted in a strain with increased hydrophobicity in culture, likely resulting from alterations to the LOS profile of the mutant strain.

### The △*mmpL12* strain is deficient in LOS export

**Fig. 5.**
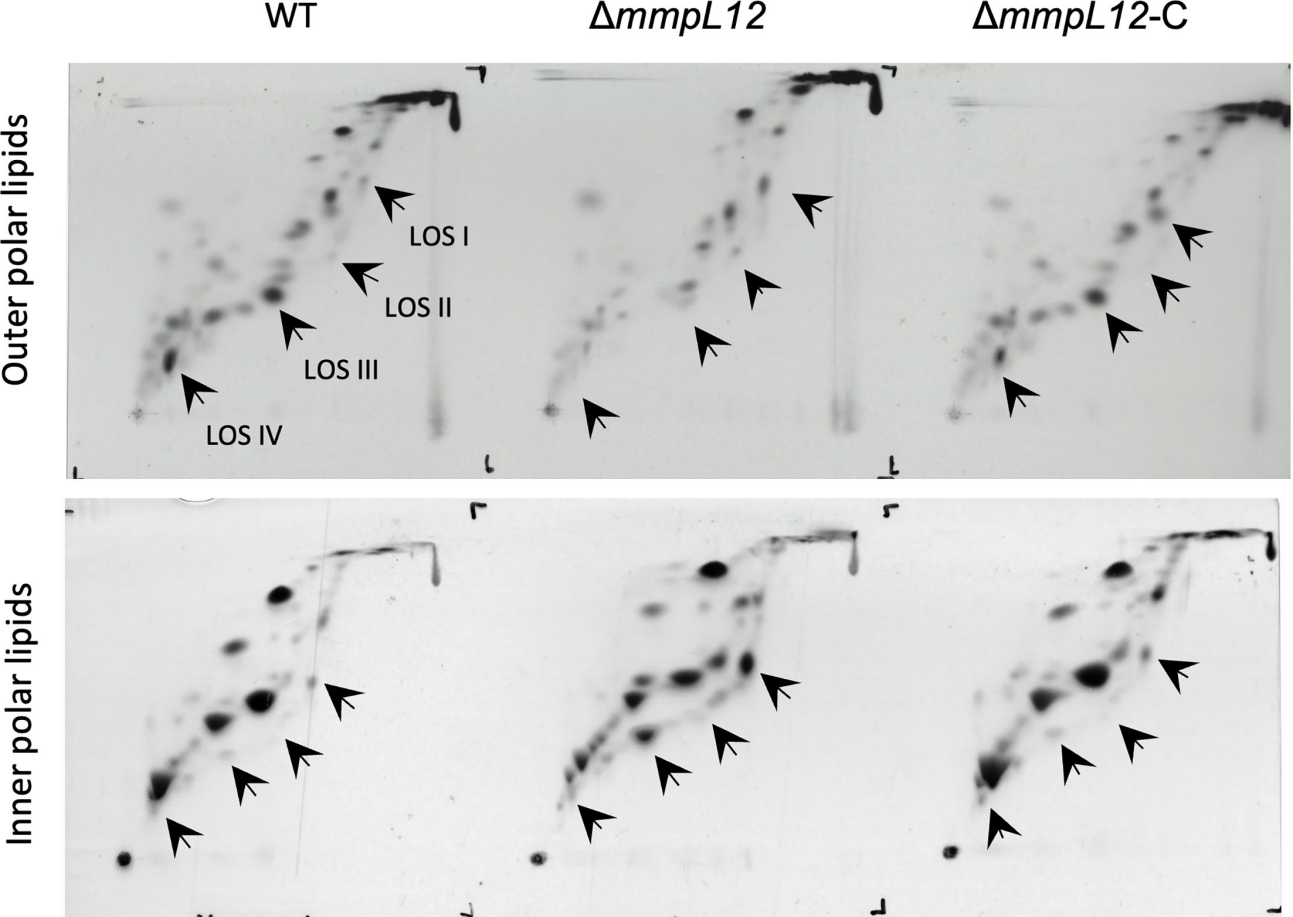
2D-TLC analysis of ^14^[C]-labelled polar lipids from *M. marinum* strains. Outer polar and apolar lipids were extracted first using water-saturated butanol from WT, mutant (Δ*mmpL12*) and complemented (Δ*mmpL12*-C) strains, followed by the extraction of inner polar lipids. Lipid species were separated using solvent system E (direction 1, CHCl3:CH3OH:H2O [60:30:6]; direction 2, CHCl3:CH3COOH:CH3OH:H2O [40:25:3 6]) and visualized on X-ray films by autoradiography. LOS species are indicated by arrows (for the mutant strain, arrows pointing to empty spots indicate putative positions where a LOS species would be expected to migrate). Set of TLCs represents one of two repeats.

To probe the reasons for altered colony morphology and increased hydrophobicity, we subjected the WT, △*mmpL12* and △*mmpL12*-C strains to lipid analysis. Cultures were grown in the presence of [^14^C]-acetate to label lipids. We first extracted ‘outer’ lipids from cell pellets of the strains using water-saturated butanol [31]. This extract would contain both polar and apolar lipid species. The butanol-treated cells were then subjected to lipid extraction methods designed to obtain both apolar and polar fractions from the cells (LOSs are present in the latter fraction). The butanol and polar lipid extracts were then analysed by 2D-TLC optimized for the separation of polar lipids, including LOSs. Previous lipid analysis of LOS-deficient mutants reported 2D-TLCs of total polar lipids (inside+outside), and thus, this approach also allowed us to query the localization of each LOS class. We used the previously described LOS migration patterns to determine the LOS species profile of the WT, △*mmpL12* and complemented strains [6]. All LOS species were found predominantly in the butanol extract for the WT strain (with LOS-III and IV being the abundant species) in the WT strain (Fig. 5). The △*mmpL12* strain however showed a number of alterations (Fig. 5). First, no LOS-IV was detected in both the butanol and the inner polar lipid fractions. Second, the mutant strain was not deficient in LOS intermediate biosynthesis and showed a clear and abundant accumulation of LOS-I, LOS-II and LOS-III in the ‘inner’ polar lipid extracts (Fig. 5). These data indicated that in the absence of *mmpL12*, *M. marinum* accumulated LOS-I, LOS-II and LOS-III intracellularly and that further glycosylation of LOS-III to LOS-IV required *mmpL12*. LOS export was restored in the complemented strain, confirming the role of *mmpL12* in the export of this glycolipid (Fig. 5). These findings confirm that *mmpL12* is required primarily for the export of LOS-III in *M. marinum*. We did not observe any defects in the transport of other (apolar) lipid species in the mutant (Fig. S1, available in the online Supplementary Material).

### Structural analysis of MmpL12 organization

To see if we may obtain any insights about the function of the *M. marinum* MmpL12 (UniProt ID B2HQ48), we modelled the protein using AlphaFold3 [32], both as a monomer and as multimer, and compared it to known MmpL structures [33,36]. As previously reported [33,37,39], MmpL12 falls within the subclass 1 (*viz* cluster 1) of MmpLs, which includes all the MmpLs except MmpL3, MmpL11 and MmpL13 (which, in themselves, form the smaller subclass 2). Both MmpL subclasses feature a common TM topology with 12 TM helices and 2 large periplasmic loops spliced between TM1 and TM2 (periplasmic domain 1, aka PD1) and TM7 and TM8 (PD2 domain), respectively (Fig. S2A). However, while in subclass 2 MmpLs, both PD1 and PD2 domains are contiguous and share the same topology as seen in the classical RND transporters [40,41], subclass 1 MmpLs feature a large insert in the PD2 domain, predicted to have a high *α*-helical content, which is sometimes referred to as PD3 [37,42]. In addition, in MmpL12, there is also a prominent C-terminal extension domain (residues ~1,003–1,457), which shares no significant homology with other known sequences. Experimental structures have confirmed that the core TM organization, as well as the PD1 domain, is shared across all the MmpLs, but there is still limited clarity regarding the function and organization of the PD3 domain and, in particular, the role of the large C-terminal extension domain.

Initially, we modelled *M. marinum* MmpL12 (UniProt ID B2HQ48) as a monomer, using the AlphaFold3 server (https://alphafoldserver.com/), which resulted in a high confidence model for the core of the structure (covering the TM and PD1/2 domains), with an average pLDDT score of 80.2 and predicted template modelling (pTM) score of 0.76, indicative of a high confidence model, but also featured a highly disordered and unstructured C-terminal segment (bringing the overall pLDDT score for the full-length MmpL12 to 64.6 and pTM: 0.60), although, notably, a segment of it (covering residue range ~H1117 R1427), featuring a high number of NG-rich repeats, was predicted to have high propensity of *β*-strand formation (Figs S2B and S7). Additional analysis of the MSA coverage and depth using AlphaFold2 as implemented in ColabFold v1.5.5 [25] indicated that the C-terminal domain lacks meaningful depth of sequence coverage and correspondingly very low PAE scores for the region (Fig. S7), so it could only be used for qualitative models (see below).

As mentioned above, the core of the MmpL12 exhibited a classical domain structure as in other RND family transporters, including AcrB and hopanoid transporters [43,44], which couple proton-motive force to substrate-extruding conformational change, via so-called proton-relays composed of conserved charged residues. A closer examination of the TM domain of MmpL12 showed high conservation of the residues which in other, well-studied MmpLs, such as MmpL3 [35,36] and MmpL4/5 [41,42] (Fig. S3), have been experimentally linked to proton dependence [45,46]. The corresponding residues in MmpL12 present two conserved pairs of aspartic acid-tyrosine (DY) residues, DY1 (D283/Y284 in *M. marinum* and D268/D269 in *Mtb*, respectively), located on TM4, and DY2 (D912/Y913 in *M. marinum* and D897/Y898 in *Mtb*, respectively), located on TM10. There is also conservation of the auxiliary residues located on TM12 (D980/R985 in *M. marinum* and D965/D970 in *Mtb*) (Fig. S3). As such, MmpL12 appears to present features associated with the classical proton-driven MmpL [46,47] unlike, e.g. MmpL7 [38].

A prominent feature of the MmpL12 model is the *α*-helical PD3 domain, which covers some 256 residues (E499–P755, using full-length MmpL12 numbering as per *M. marinum* UniProt ID B2HQ48), and in the monomeric model, it folds into itself, resembling a Swiss-army knife (hinging at residues 573 and 668, respectively), which limits its length to ~108 Å. At the tip of the folded hairpin, there are two very closely positioned conserved cysteines (C623-C629), the C*α*-atoms of which are within 5.3 Å in the model, suggestive of a potential intramolecular disulphide formation (Figs S2 and S4). Surprisingly, when MmpL12 was modelled as a trimer, the PD3 was found to assume an extended conformation, forming a six-helical tubular coiled-coil trimerization scaffold that extends to ~180 Å into the periplasm. In this arrangement, the conserved cysteine residues are positioned at the very tip of the extended tubular extension (Figs 2 and S5). This was also the case for the MmpL12 from *Mycobacterium bovis* BCG and the MmpL12 from *M. tuberculosis*. All AlphaFold3 modelling has been done in triplicate, with a different seed per run and five predictions per seed. Model quality and statistics are shown in Fig. S8. Overall pTM and interface predicted template modelling (ipTM) scores are between 0.52 and 0.60 and 0.45 and 0.55, respectively, indicative of moderate model confidence; however, the model confidence scores are very unevenly distributed between the individual domains, with the TM section showing significantly higher pTM and pLDDT scores (pTM: 0 . 72; ipTM: 0.62 for the top MmpL12 *M. marinum*) than the coiled-coil domain and particularly the C-terminal domain, reflecting the limited depth of MSA coverage and depth in this region as discussed previously (Fig. S7 and associated PAE matrix).

As MmpL12 is phylogenetically more distantly related to other subclass 1 MmpLs [48], and to see whether the predicted trimerization may hence be limited to it alone, we also modelled, apart from its closest MmpL12 relatives from *M. bovis* BCG and *M. tuberculosis,* a full complement of *M. tuberculosis* subclass 1 MmpLs (covering MmpL1-2 and 4–10) and *M. marinum* MmpL1, MmpL2, MmpL4 and MmpL7. Notably, all trimeric structures for the above MmpLs were generated with very high confidence (AlphaFold3 ipTM and pTM scores over 0.75 and 0.80, respectively, across the group) and represent highly similar predicted structures with an overall RMSD of under 1 Å (Figs S5, S6 and S8).

Indeed, *Mtb* MmpL1 (P9WJV9), MmpL2 (P9WJV7), MmpL4 (P9WJV3), MmpL5 (P9WJV1), MmpL6 (Q8RK80), MmpL7 (P9WJU7) and MmpL9 (P9WJU3) have been suggested to be most closely associated [48], confirming that they all exhibit very closely related helical extension (see Fig. S5B), which in a trimeric configuration reaches about 120–125 Å into the periplasm (and a total length of about 140 Å) (Figs S5C-D, S6 and S8), which allows to group them as a subclass 1a. However, both MmpL8 (P9WJU5) and MmpL10 (P9WJU1) showed a distinct organization, most closely aligned to the MmpL12 (B2HQ48 for *M. marinum*, P9WJT for *Mtb* and A0A0H3M689 for *M. bovis* BCG) (Figs 2, S4, S6 and S8). In comparison to the *M. marinum* MmpL1 (A0A2Z5YCA0), the *α*-helical domain of MmpL12 in *M. marinum*, *Mtb* and *M. bovis* appears to be more complex and resembles closest the *α*-helical domain of MmpL8 and MmpL10 (Figs S3, S4 and S6). In *M. marinum*, the PD3 hairpin is predicted to cover ~260 residues (E458-P719 and E502-P763, respectively) and it shows a higher level of homology.

When modelled using AlphaFold3, MmpL12, 10 and 8 (from here referred to as subclass 1b) show very similar structure of the TM, PD1-2 and PD3 domains, with the PD3 assuming an extended trimeric tube configuration, which is noticeably longer than that of the PD3 domains in the subclass 1a MmpLs, with the former being ~180 Å in length and protruding some 170 Å from the crown of the PD1/PD2 domains, with the tip being up to 210 Å from the membrane plane. For the MmpLs of subclass 1a, these numbers are significantly lower, at ~125 Å for the PD2 and a total extension from the membrane of up to 170 Å (Figs S6 and S8).

Despite sharing a close homology to the MmpL8-10-12 subcluster of *Mtb*, the *M. marinum* MmpL12 possesses a unique C-terminal domain, covering ~450 residues (1,003–1,457 residues), which shows no significant overall homology to known sequences and contains a high percentage of low complexity regions, making it difficult to model. Subsequently, it is shown as unstructured in the monomeric model of MmpL12 (Figs S2 and S7). However, when modelled in isolation, a section of the C-terminal domain (residues H1117-R1427) displays high propensity for *β*-strand formation and, strikingly, when modelled in AlphaFold3, consistently presents as a compact trimeric right-handed *β*-helical assembly (Figs 2 and S9A), which may provide additional nucleus for oligomerization and/or provide scaffolding for additional factors.

The remainder of the C-terminal domain does not fold into a defined structure when modelled on its own, but when MmpL12 is modelled as a trimer, it presents a split globular domain with high *α*-helical content, formed out of residue ranges P1003-G1156, and then again from approximately S1428 to the C-terminal residue R1457, although as mentioned earlier, the modelling confidence is very low for the C-terminal region. We therefore used these separate modelling outputs to assemble a composite qualitative model of the complete trimeric assembly of *M. marinum* MmpL12 (Fig. 2). To our knowledge, this is the first complete model of *M. marinum* MmpL12, which also provides a general rationale to the trimerization of the subclass 1 MmpLs as a whole.

In summary, our modelling suggests that MmpL12 is likely a proton-driven pump within subgroup 1 of MmpLs. It also suggests that both subgroup 1a MmpLs (covering MmpL1, 2, 4–7 and 9) and subgroup 1b (MmpL8, 10 and 12) use their respective PD3 domains for trimerization, allowing them to span the periplasm and anchor/deliver substrates to the mycobacterial outer membrane; however, the respective PD3 domains present different unique characteristics in both subgroups.

### Absence of LOS export impacts *M. marinum* virulence

**Fig. 6.**
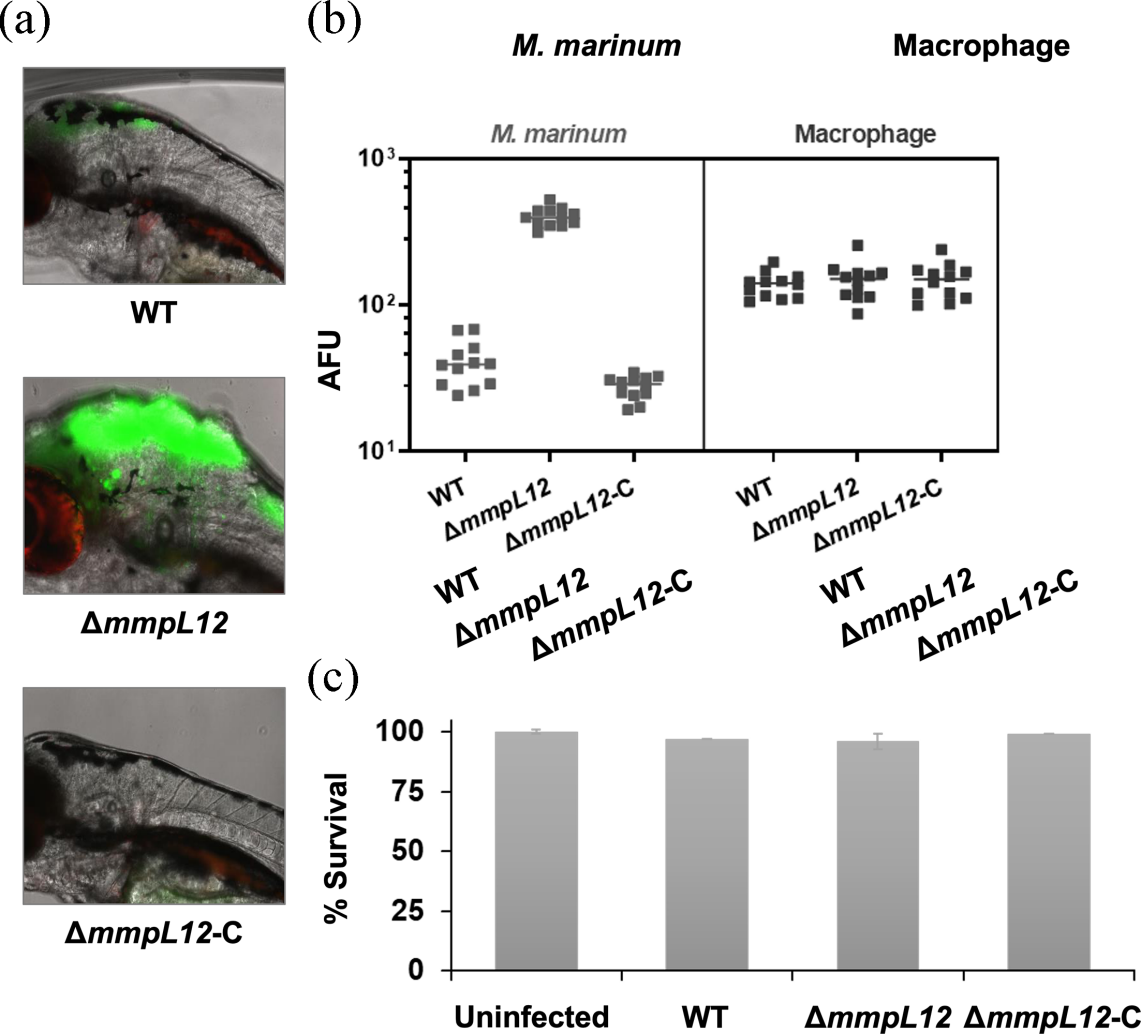
The absence of exported LOS impacts *in vivo* growth. (**a**) Representative images of 4 dpi zebrafish larvae (*n*=50) that were infected with about 200 c.f.u. of the indicated *M. marinum* strains. (**b**) Bacterial burden and macrophage response (4 dpi) are represented by the green and red pixel counts, respectively, from the fish larvae (*n*=12) that were infected with the indicated *M. marinum* strains. AFU indicates arbitrary fluorescence units. (**c**) The survival of the fish larvae (4 dpi) infected with the indicated *M. marinum* strains. Survival of uninfected control fish larvae was considered 100%.

As rough mycobacterial variants are known to exhibit enhanced virulence [4], we investigated whether the lack of exported LOSs and consequent rough-colony morphology results in enhanced virulence in its natural host, zebrafish. We employed a zebrafish line with macrophage-specific mCherry fluorescent protein expression. One-day-old embryos of this line were micro-injected with *M. marinum* WT, *ΔmmpL12* and *ΔmmpL12*-C strains, all expressing mWasabi fluorescent protein (Fig. 6a). This combination enabled us to monitor the bacterial infection (mWasabi) and the host macrophage response (mCherry). The fish that were infected with the knock-out strain, *ΔmmpL12,* exhibited significantly higher levels of bacterial burden than those infected with the WT or with the complemented strain, *ΔmmpL12*-C. The embryos infected with either WT, *ΔmmpL12* or *ΔmmpL12*-C strains exhibited comparable macrophage response (Fig. 6b) and survival rates (Fig. 6c). These observations suggested that the absence of the exported LOSs in the *ΔmmpL12* strain resulted in enhanced replication of the mutant strain in zebrafish embryos.

## Discussion

LOSs are part of an array of complex lipids found in the cell envelopes of mycobacteria. These complex lipids are primarily transported by the RND family MmpL proteins [10,11]. Some lipids, such as phthiocerol dimycocerosate, are synthesized completely on the cytosolic side and transported by MmpL7 [49]. Transport of sulfolipids, on the other hand, occurs via the transport of a diacylated intermediate, which subsequently gets acylated after transport by MmpL8 [50,51]. Similarly, di- and penta-acylated trehaloses (DAT and PAT) require MmpL10-mediated export of DAT, which is subsequently acylated on the outside of the cell to yield PAT [52,53]. The genetic cluster for LOS biosynthesis contains many genes encoding glycosyl transferases similar to eukaryotic dolichol phosphate mannose synthases. These enzymes are members of the GT-2 family of glycosyltransferases (GTFs) that catalyse the transfer of sugars to dolichol phosphate by using nt sugars as substrates. In mycobacteria, members of this family of GTFs use polyprenol rather than dolichol, forming lipid-anchored sugar precursors that act as sugar donors for extracellular GTFs. We reasoned that the presence of such GTFs in the LOS cluster could indicate that LOS biosynthesis begins in the cytoplasm but is completed extracellularly [54]. Thus, MmpL12 would likely transport an early LOS intermediate. Analysis of cytosolic and outer cell envelope fractions of the △*mmpL12* mutant revealed a loss of LOS-IV and intracellular accumulation of LOS-I, LOS-II and LOS-III, suggesting that MmpL12 was capable of LOS-I, LOS-II and LOS-III export, but LOS-IV was synthesized by further glycosylation of LOS-III after its export by MmpL12. Thus, LOS biosynthesis is likely initiated intracellularly, but LOS-IV is generated extracellularly after MmpL12-mediated LOS-III export. Thus, like other MmpLs, MmpL12 may also act as a scaffold for LOS biosynthesis enzymes, particularly GTFs [55].

The absence of LOS export resulted in a distinctive phenotype in infected zebrafish embryos: the △*mmpL12* strain was hypervirulent and proliferated significantly more in the infected embryos. In *M. marinum*, LOSs play a key role in macrophage entry with defects in LOS biosynthesis, resulting in increased host cell entry (albeit with an increased clearance by cultured macrophages) [56,57]. Additionally, LOSs inhibited the secretion of TNF-*α* in human macrophages [8]. Loss of LOS export has been suggested to have played a role in the evolution of *M. tuberculosis* as a successful global pathogen, from an LOS-producing *M. canetti*-like ancestor. In support of this hypothesis, smooth to rough colony transition in *M. canettii* is related to the loss of LOSs, and rough strains showed an increased virulence in cell culture and animal models of infection [4]. A similar association has also been observed in *M. kansasii* [58]. It has been hypothesized that LOSs may mask other virulence-associated outer envelope lipids and that exposure of these lipids may lead to enhanced virulence in LOS-deficient mycobacteria [58]. Interestingly, an *M. marinum* mutant defective in the biosynthesis of LOS-IV also displayed a hypervirulent phenotype: the *wecE* mutant, which failed to make LOS-IV and accumulated LOS-III instead, caused increased bacterial loads and early granuloma formation in infected zebrafish embryos [59]. We cannot conclude whether the hypervirulence observed in the *mmpL12* mutant was due to defective localization of LOSs, or specifically due to the absence of LOS-IV. The *ΔmmpL12* strain exhibited high aggregation phenotype and thus clogged the needles used for systemic caudal vein infections. Therefore, despite our best efforts, we could not prevent the needle clogging, which prevented us from studing the role of MmpL12 in systemic infection.

However, the lack of any externalized LOSs makes the former more likely. While our studies showed that *mmpL12* was required for LOS export, the phenotype of the mutant also sheds light on the potential steps of LOS biosynthesis in *M. marinum*, suggesting that LOS biosynthesis initiates intracellularly but is completed by the generation of LOS-IV extracellularly.

Recent years have led to significant advancement in our understanding of MmpL structure; however, the organization of their PD3 domains and even their oligomeric state remain a subject of debate. Our modelling suggests that similar to the bacterial RND efflux pumps and the closely related transporters such as CmpL1 [60,61], subgroup 1 MmpLs are likely to form trimers. This is contrary to the subgroup 2 MmpLs (including MmpL3, 11 and 13 a/b [37]) which, as reported by a number of experimental structures and biophysical analyses, decisively form dimers [36,62,64].

Importantly, our modelling provides a structural rationale for the previously reported trimerization of the subgroup 1 MmpLs, based on single-molecule studies of MmpL5-MmpS5 [65]. We propose that the PD3 domains are central drivers of trimerization. The predicted length of the trimerized coiled-coil PD3 domains makes it possible to span the periplasm across to the mycolate membrane and removes the need for additional adaptors (outer membrane factors), which have been previously proposed [39], but have not been identified in the mycobacterial genome. While very recent works by Malmsheimer *et al*. [66], followed closely by Earp *et al*. [41], indicated that the PD3 helical coiled-coil in MmpL10 and MmpLs 4/5, respectively, protrudes far into the periplasm, these studies stopped short of providing a connection between the PD3 structure and its potential role in trimerization. Importantly, a recent pre-print which has become available while this manuscript has been under revision seems to provide experimental validation to our predictions of trimerization of the PD domains [67].

Our modelling also sheds light on the organization of the unique C-terminal extension in MmpL12, observed in *M. marinum,* which is predicted to be a low complexity region in its orthologues. In *M. marinum*, an NG-rich segment of it shows propensity to adopt a *β*-helical conformation. There are no direct sequence homologues to the NG-rich section of the MmpL12; however, a *β*-solenoid structure is predicted to be adopted by the similarly NG-rich repeats observed in curli subunits [68], while one of the top DALI server search matches and highest sequence identity (17% identity) template corresponds to the crystal structure of the hyper-glycosylated fragment of the autotransporter TibA passenger domain (4Q1Q.pdb [69]), revealing 35 heptose conjugates forming patterned and solenoid-like arrays on the surface of a *β*-helix (Fig. S9B). Thus, it is plausible that the solenoid structures predicted for the C-terminal domain of MmpL12 may be used as a scaffold for substrate concentration and/or processing. Finally, a number of bacterial enzymes, including a wide group of transferases belonging to the left-handed *β*-helix superfamily, such as the *N*-acetyltransferase PerB (4EA8.pdb [70]) and the tetrahydropicolinate succinyltransferase (3R8Y.pdb), use analogous *β*-helical structures for trimerization (Fig. S9C). Similar assemblies are found in the trimeric LpxA-like enzymes, and intriguingly, the Rv1505c (UniProt ID P71784), which is predicted to interact with MmpL12 [71] in *Mtb* H37Rv, belongs to this family (Fig. S9D), suggesting that perhaps such trimeric scaffolds are provided by an auxiliary protein in MmpLs that lack the NG repeats. While speculative, these observations suggest that the C-terminal domain of MmpL12 may play a role in supporting the trimerization of the transporter and its association with other enzymes within the biosynthetic pathway and provide a testable hypothesis for the MmpL oligomerization and function for the future.

## Supplementary material

10.1099/mic.0.001618 Uncited Supplementary Material 1.

## Abbreviations

dpi: days post-infection
GTFs: glycosyltransferases
hpf: hours post-fertilization
ipTM: interface predicted template modelling
LB: Luira-Bertani
LOS: lipooligosaccharide
MSA: multiple sequence alignment
PAE: Predicted aligned error
pTM: predicted template modelling
PUM buffer: Phosphate Urea Magnesium
RND: resistance-nodulation-division
TM: transmembrane
