# Supplementary material for: MmpL12 transports lipooligosaccharides and impacts virulence in Mycobacterium marinum
Source: Microbiology (Reading). 2025 Oct 8;171(10):001618. doi: 10.1099/mic.0.001618 (PMC12507525; doi:10.1099/mic.0.001618)

Fig. S1

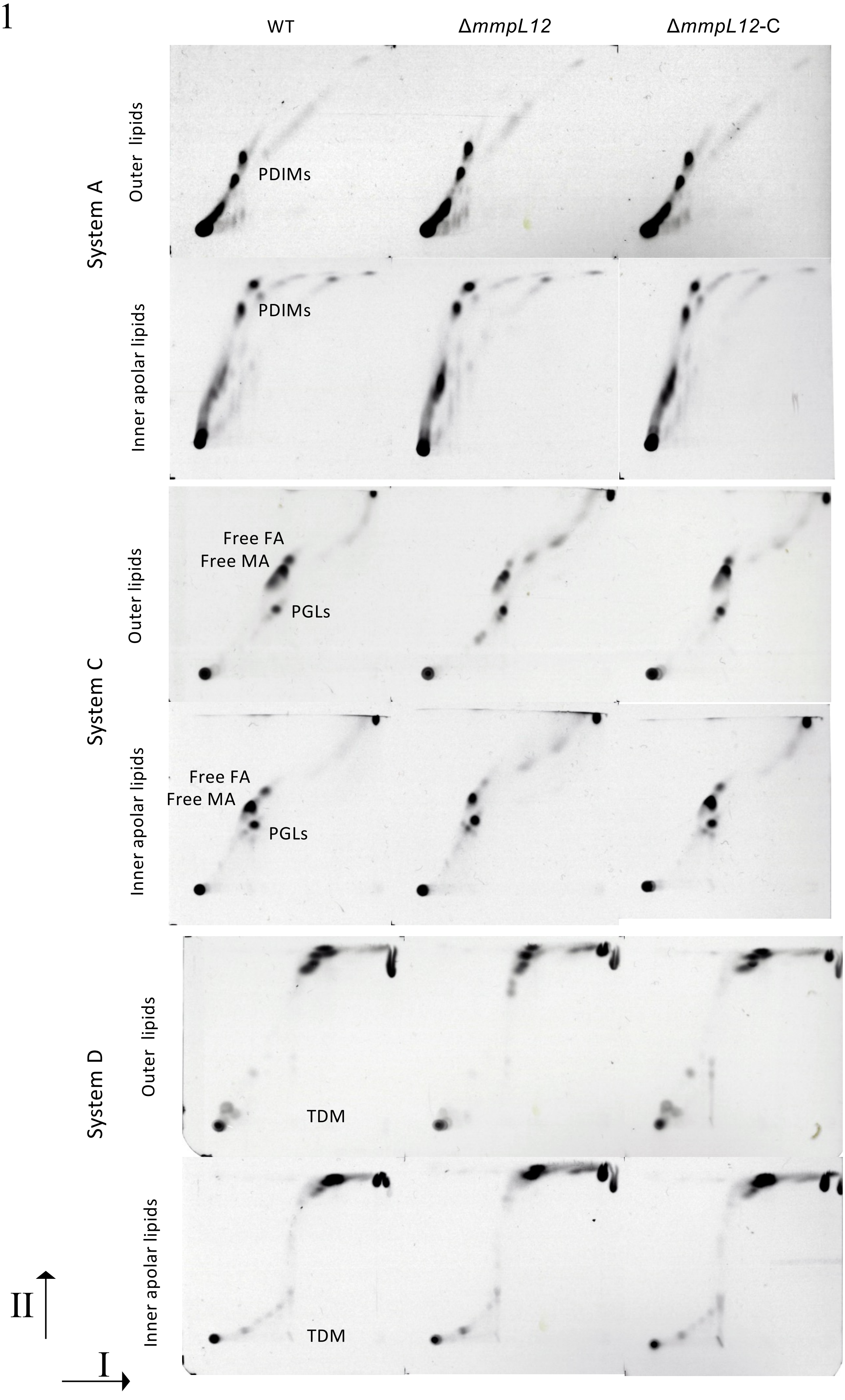
WT
ΔmmpL12
ΔmmpL12-C
System A
Outer lipids
PDIMs
Inner apolar lipids
PDIMs
System C
Outer lipids
Free FA
Free MA
PGLs
Inner apolar lipids
Free FA
Free MA
PGLs
System D
Outer lipids
TDM
Inner apolar lipids
TDM
II
I

Fig. S2

M. marinum MmpL12 monomer model

A. Topological diagram of the MmpL12 organisation, which is representative of subclass I MmpLs.

B. AlphaFold 3 model of the monomeric MmpL12, highlighting the main structural features.

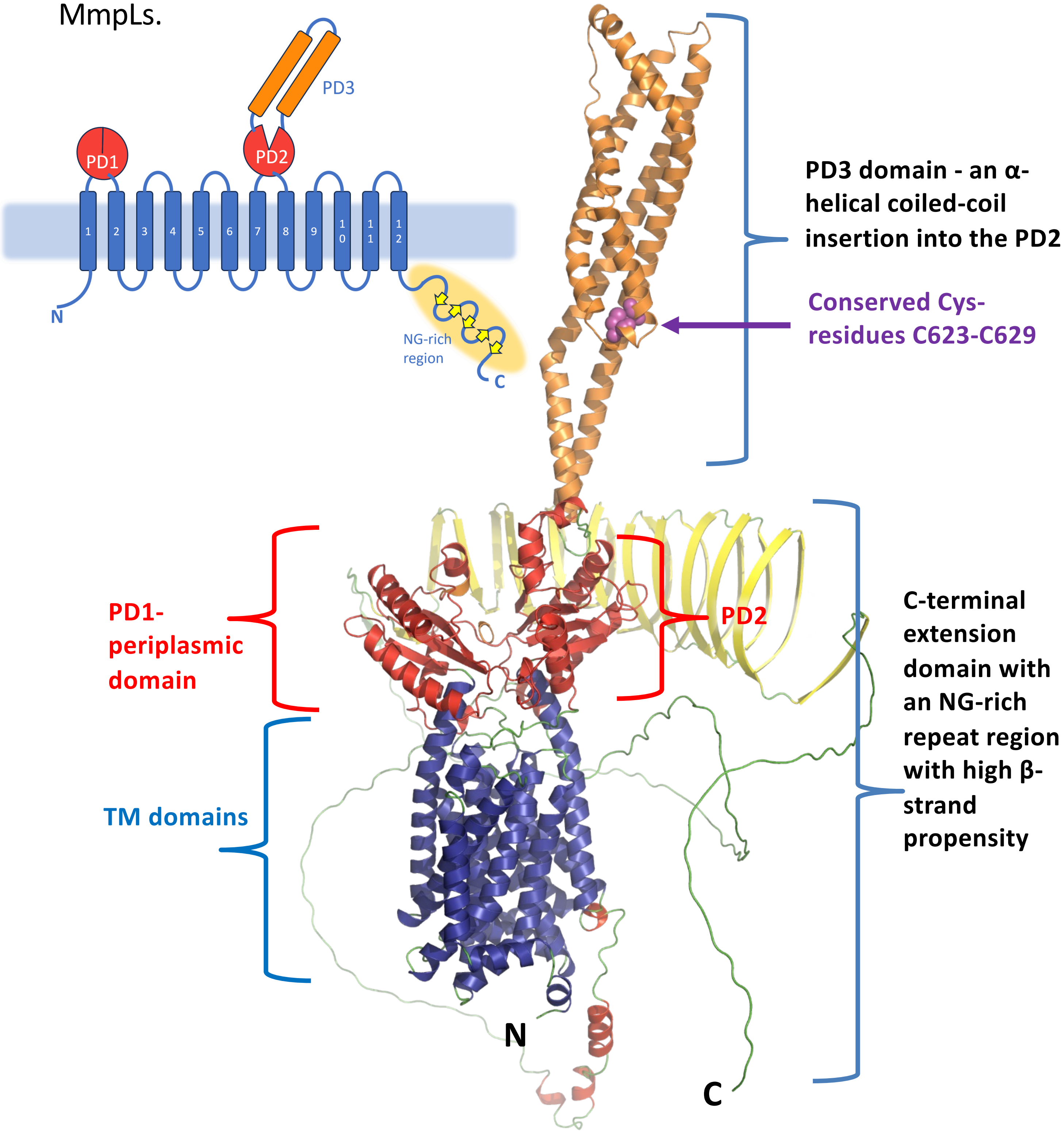

Fig S3.

Core domains and proton relays.

B. A close-up showing the conservation of the proton-relay residues discussed in the main text using the MmpL12 model (blue) and the experimental MmpL3 structure 7NVH.pdb (magenta).

B. Superposition of MmpL12 model (blue) and the experimental MmpL3 structure 7NVH.pdb (magenta). Magnified area shown in A is highlighted in yellow.

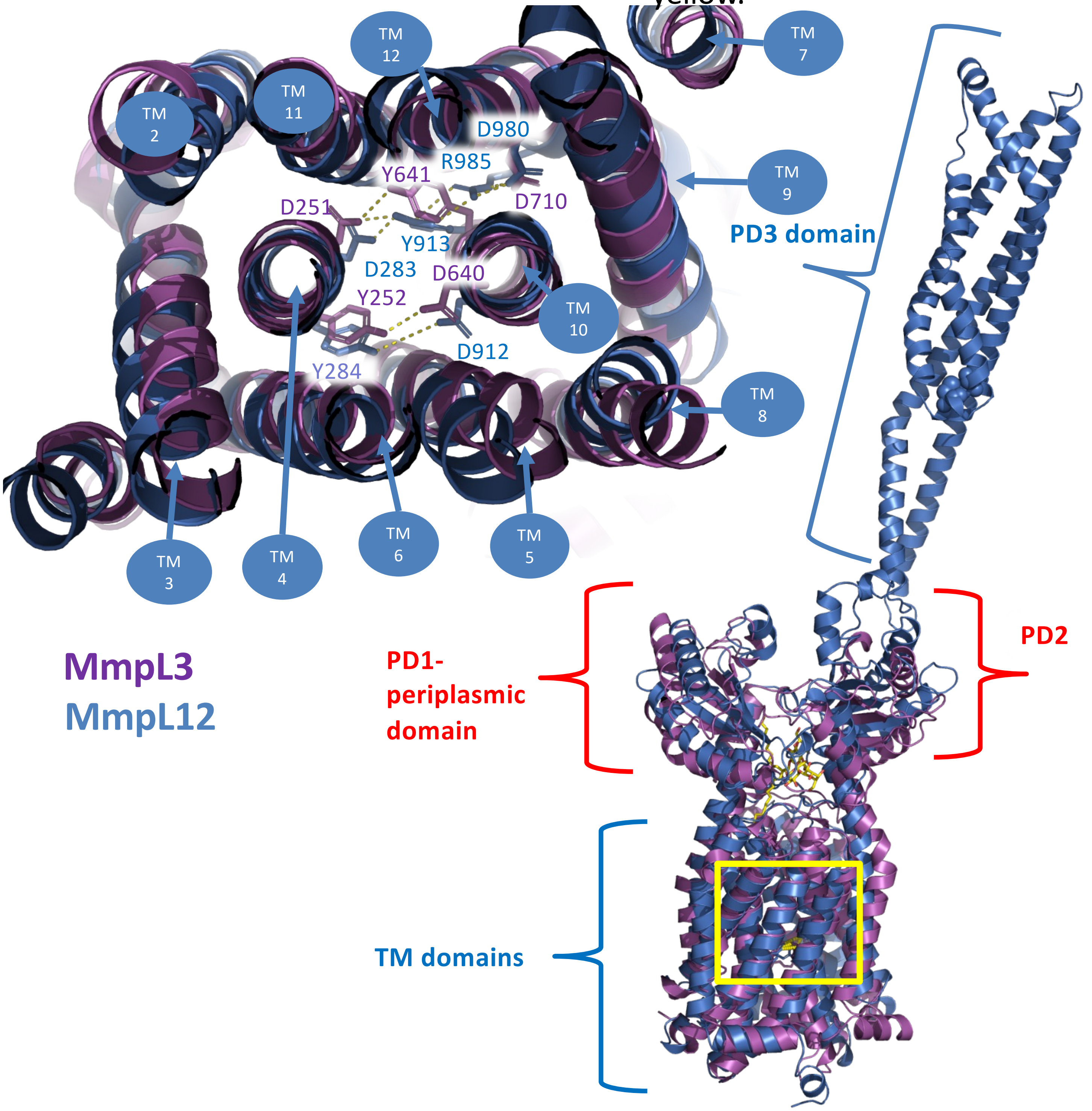

Fig S4.

M. marinum MmpL12 PD3-domain vs the corresponding PD3 domains in the closest related MmpLs.

Position of the conserved cysteines is indicated with a green C.

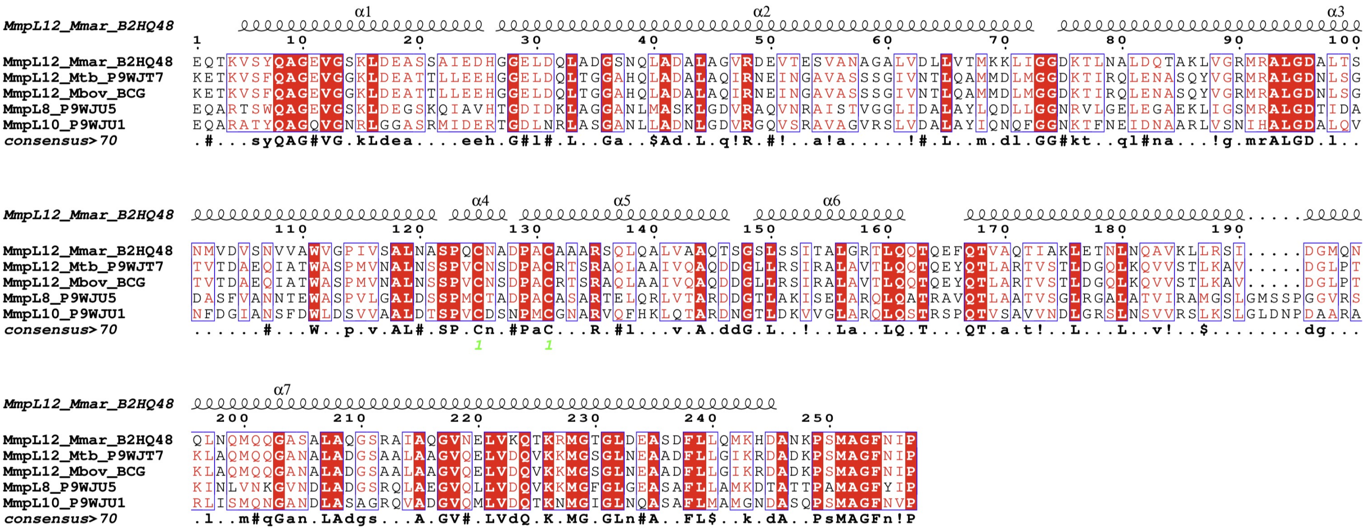

Fig S5.

Modelling of the PD3 domains across the MmpLs belonging to subclass 1, suggests that PD3 domains may promote trimerisation, and reveals two distinct subfamilies, with different predicted lengths of the hairpin.

A

Two representative subclass 1 MmpLs: Left, MmpL1 *M. marinum* (B2HPY6) & Right, MmpL7 *M. marinum* (B2HIL9); Showing very similar organisation

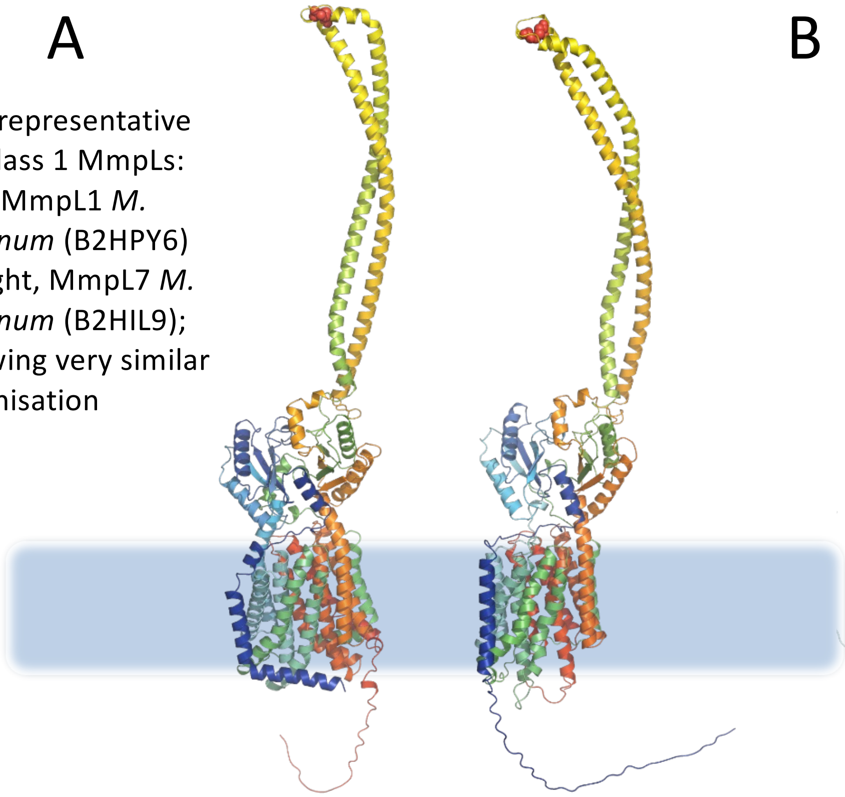

B

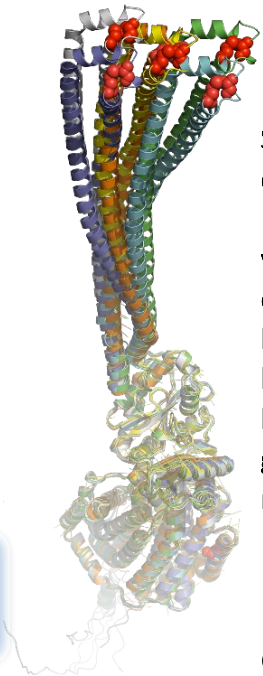

Superposition of the different subclass 1 MmpLs showing virtually the same organisation and length of the PD3 hairpins. MmpL1 – light grey; MmpL2 – green, MmpL2 M. marinum – cyan; MmpL4 – yellow, MmpL6 – blue, MmpL9 – orange. (MmpL5 and 7 omitted for clarity).

C

C. A trimeric model of *Mtb* MmpL9, showing the tubular trimerisation of the PD3 domain

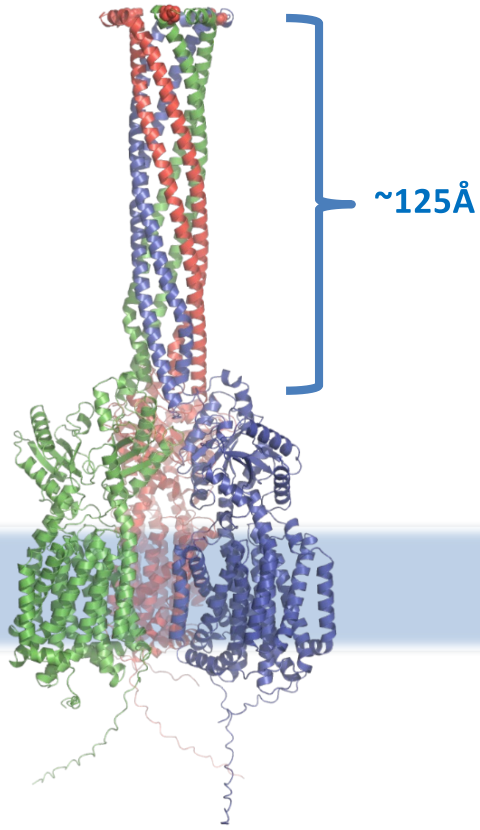

D

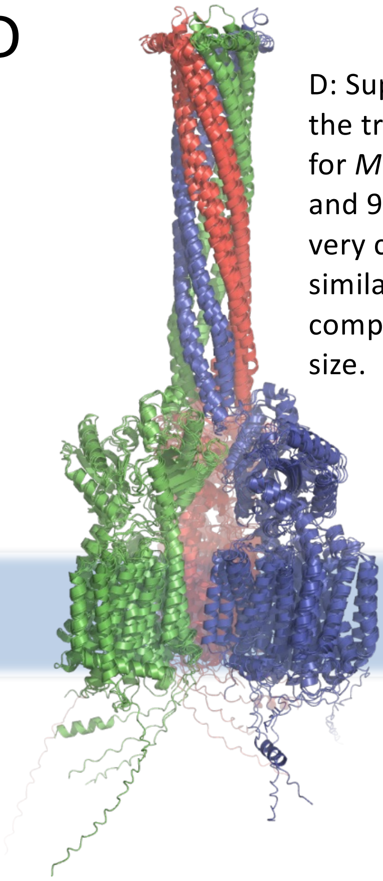

D: Superposition of the trimeric models for *Mtb* MmpL1, 5, 7 and 9, showing their very close overall similarity and comparable PD3 size.

Fig S6.

Comparison of the MmpLs from subclass 1a (1,2, 4-7 and 9) vs MmpLs from subclass 1b (12, 10 and 8 respectively), showing the different scale of the PD3 domains. The trimerisation PD3 domain is predicted to extend approximately 125 Å in subclass 1a MmpLs and up to ~170 Å when measured from the membrane plane, and around 170 Å in subclass 1b, with a total extension from the membrane to the tip of around 210Å.

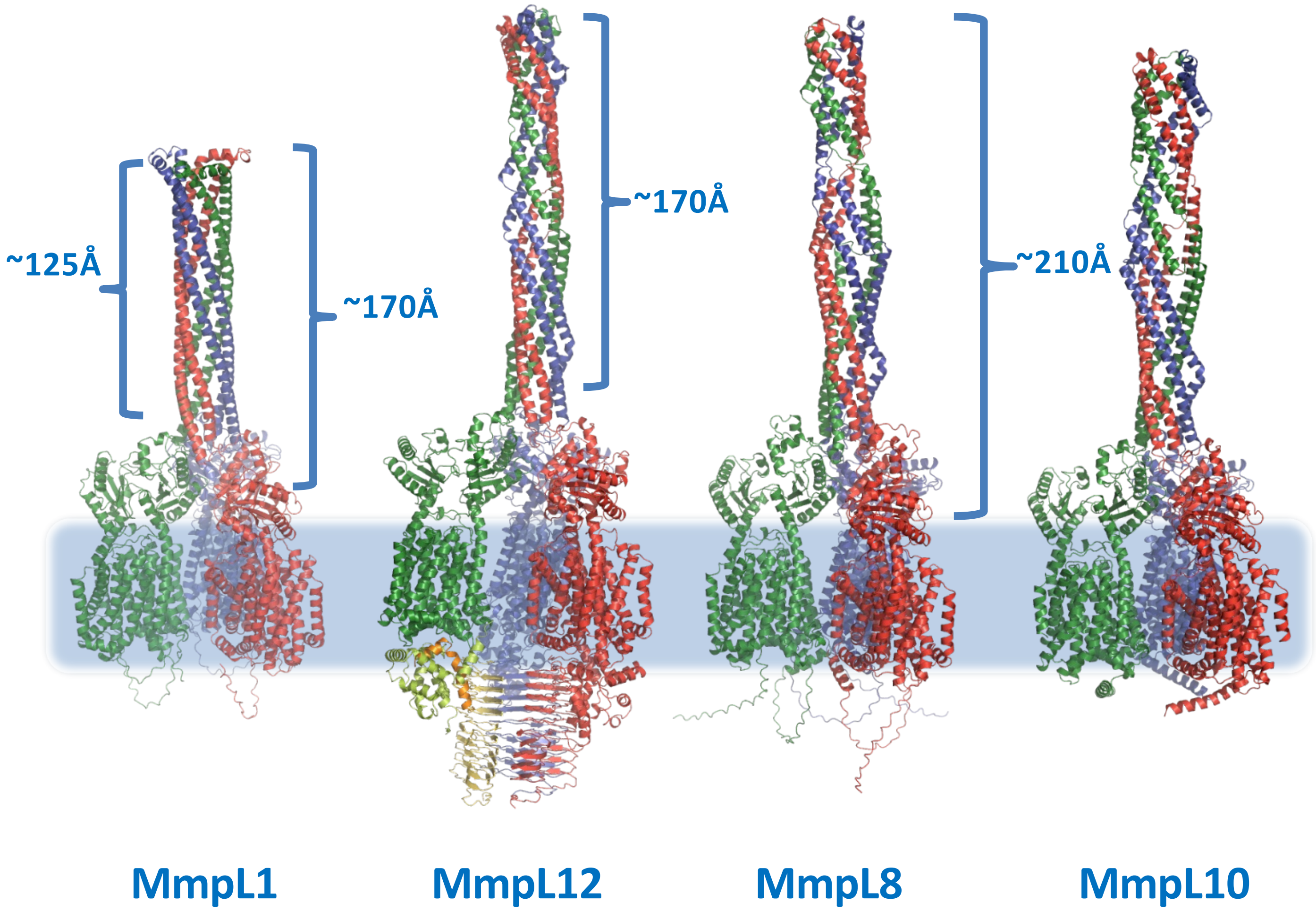

Fig S7.
MSA depth and coverage for MmpL12 *M. marinum*.

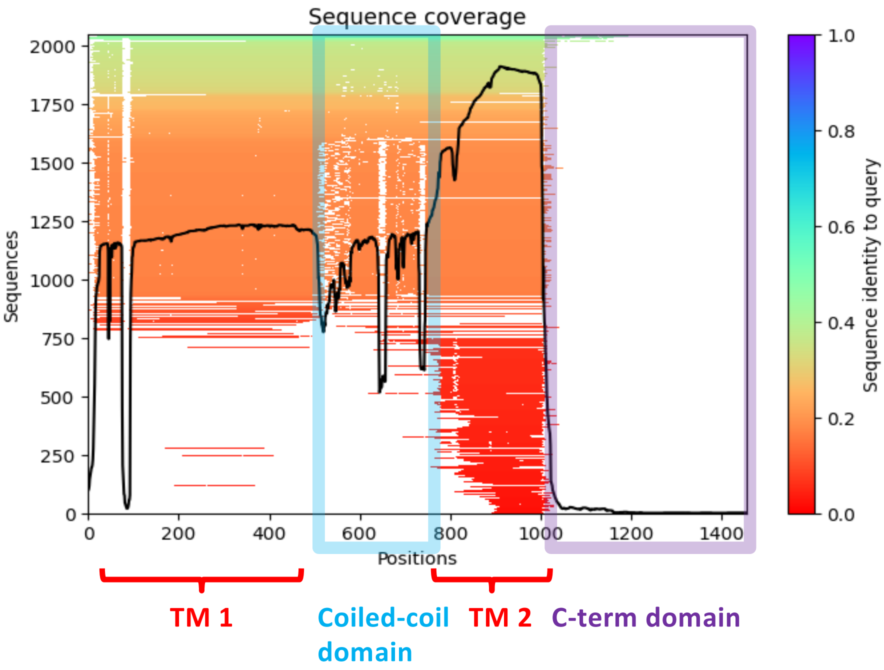

The **maximum MSA depth** (aka **full-sequence coverage line**) is the black line representing the **total number of sequences in the multiple sequence alignment** that could possibly cover a given position.
Do note the much higher conservation of the residues 750-1025, which correspond to the second lobe of the TM domain (**TM2**), housing the "proton relay" residues. Please note the lack of coverage for the C-terminal domain (highlighted by the **purple box**) and the less dramatic drop in sequence coverage for the coiled-coil domain (**blue box**) which makes their reliable modelling problematic. Due to extremely low MSA depth for the C-terminal domain, and correspondingly low pLDDT scores (<40) only qualitative models can be derived for it. Images generated by ColabFold v1.5.5: AlphaFold2 using MMseqs2 (Mirdita et al., 2022).

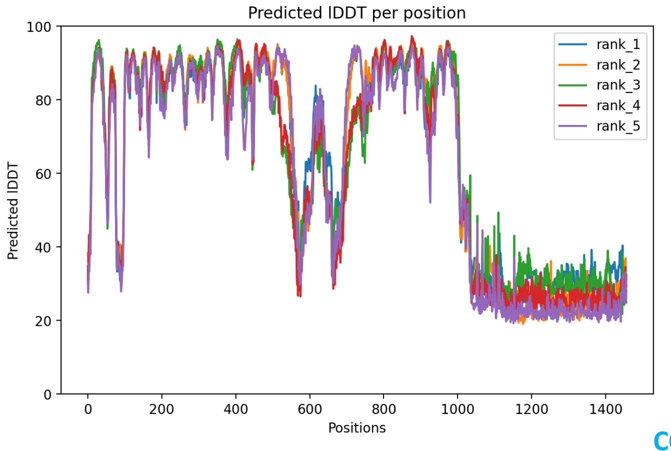

pLDDT: 64.6 (80.2)
pTM: 0.60 (0.76)

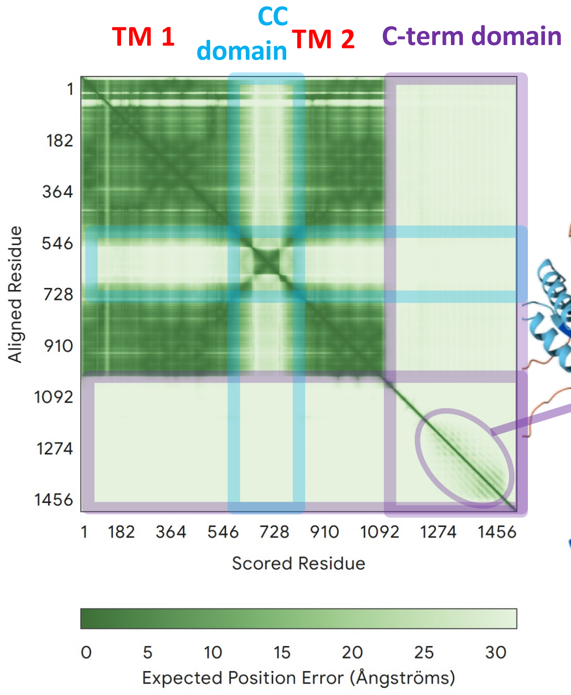

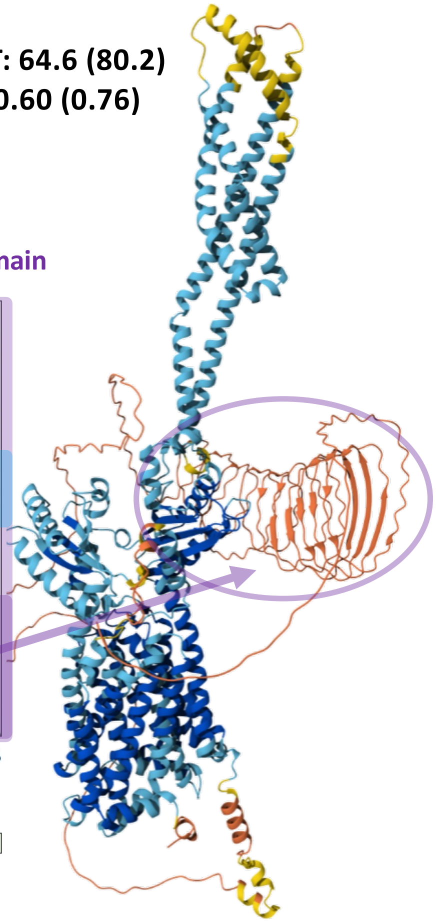

The **PAE matrix (right, green) for the MmpL12 *M. marinum* monomeric model (far right), coloured by pLDDT scores.**
The large areas of low PAE scores correspond to the C-terminal domains, and to the elongated coiled-coil region, reflecting the positional uncertainty of the residues in the respective ranges.
Note the predicted β-strand-rich region in the C-terminal region (light purple ellipse).pLDDT and pTM figures are shown for the full length MmpL12 and those in brackets correspond to the model without the C-terminal domain (1-1003), highlighting the highly disordered nature of it.

Fig S8. Model quality indicators for the AlphaFold3 prediction of MmpL12 and related MmpLs discussed in the text. MmpL12 *M. marinum*. The model with the higher ipTM and pTM scores is shown out of three technical repeats. C-terminal domain is omitted due to low fidelity. On the left, coloured by protomer, on the right by pLDDT score.

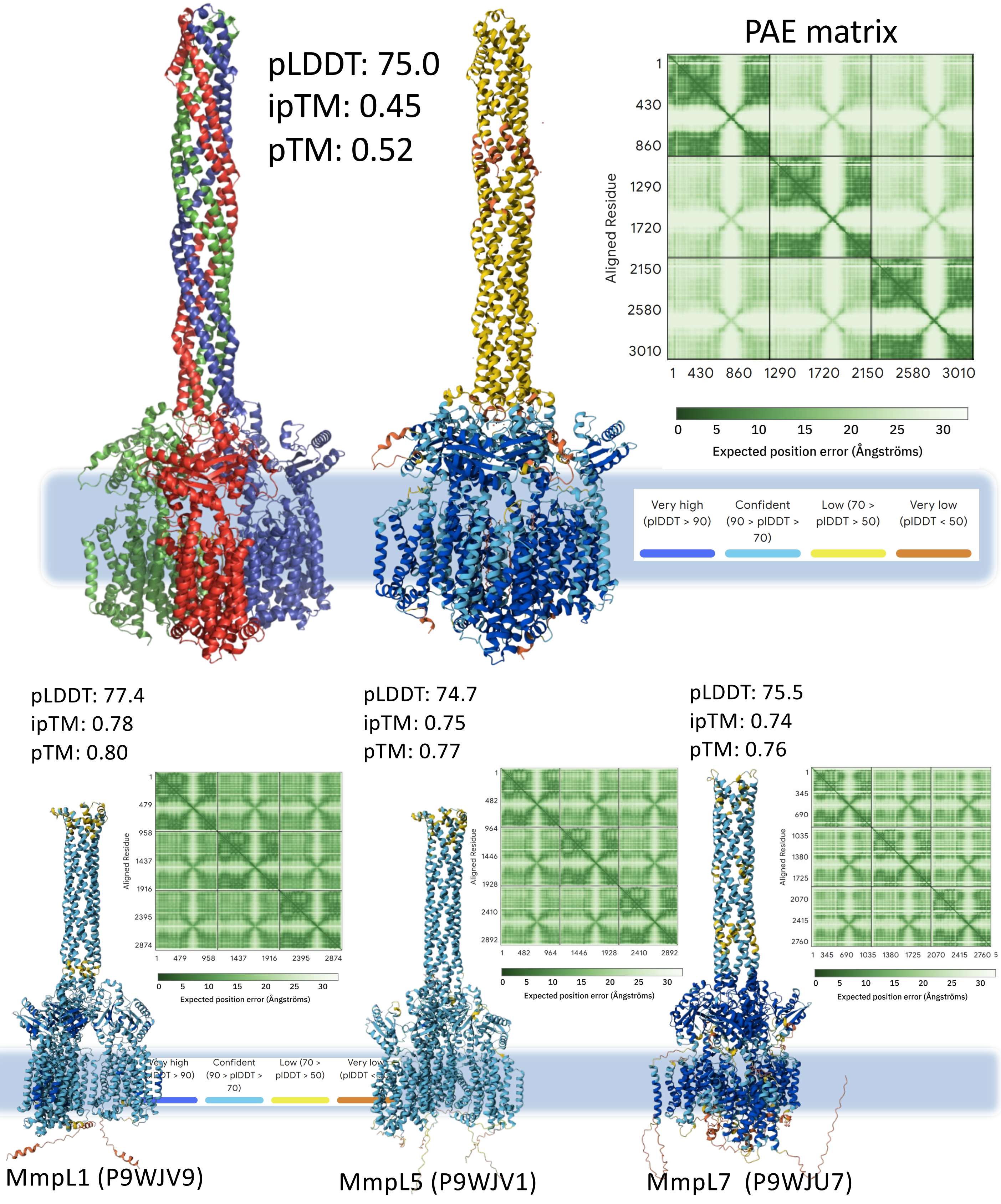

Fig S9.

A. The NG-rich section of the C-terminal domain of MmpL12 (residues 1117-1427 in *M. marinum*) may participate in trimerisation an provide a scaffold for substrate binding. **B.** A hyper-glycosylated TibA55-350 fragment (4Q1Q.pdb) which has the highest sequence identity to the MmpL12 NG-rich motif (17%), also presenting a right-handed β-helix. **C.** An analogous trimeric β-helical assembly being used as a scaffold for substrate binding in the sequentially non-related PerB (**4EA8.pdb**), a member of the left-handed β-helix family (LβH) of N-acetyltransferases in complex with GDP-N-acetylperosamine and Acetyl-CoA. **D.** AlphaFold model of Rv1505c trimer.

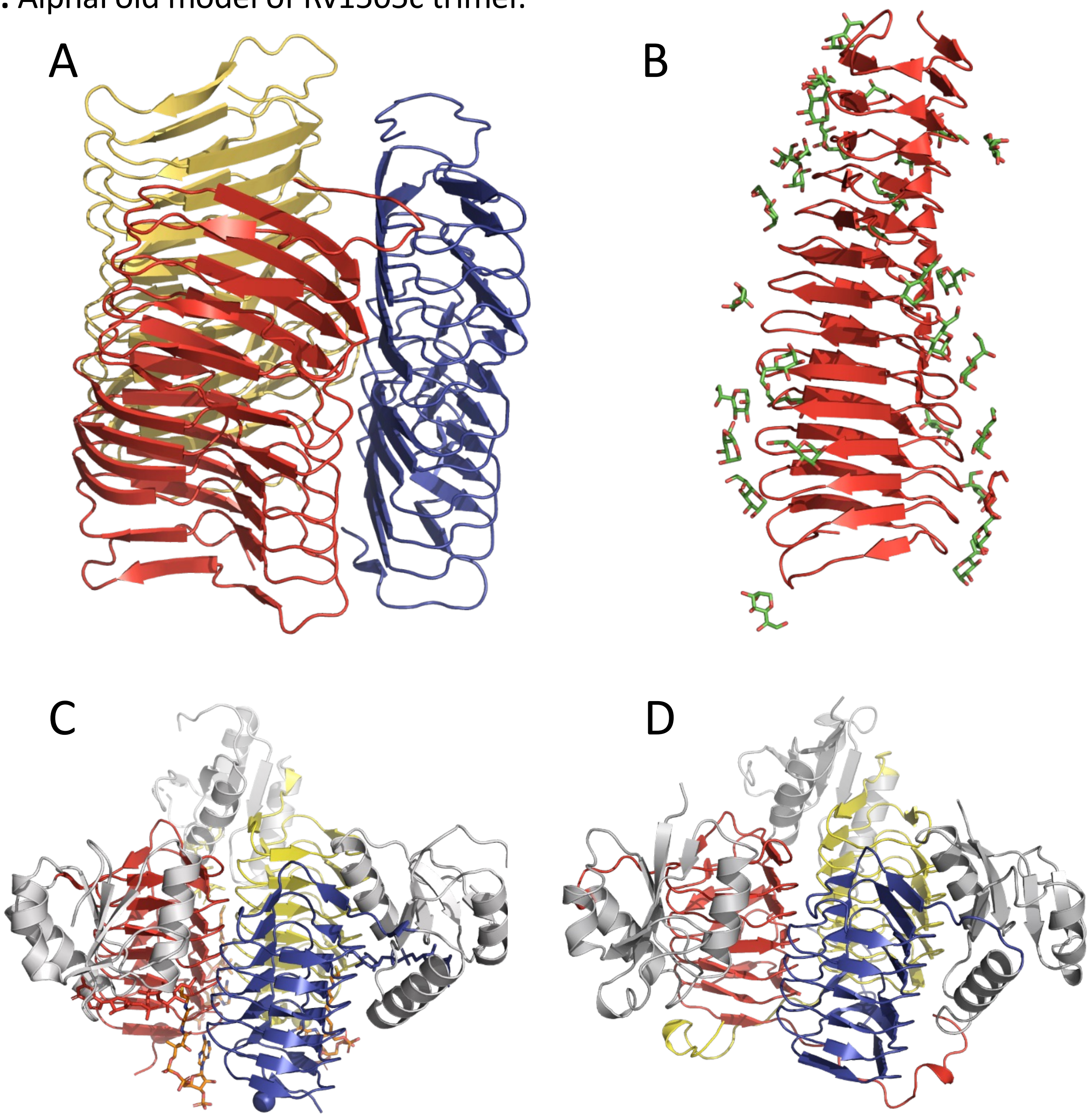

Supplement: Uncited Supplementary Material 1. [file mic-171-01618-s001.pdf]
